# Effects of fermented wheat bran on growth performance, nutrient digestibility and intestinal microbiota of weaned piglets

**DOI:** 10.3389/fvets.2025.1561196

**Published:** 2025-04-16

**Authors:** Ninghui Jia, Jin Jin, Xinru Wei, Massimo Trabalza-Marinucci, Gang Jia, Qiang Zhou, Ruinan Zhang, Hua Li, Fali Wu, Hua Zhao, Hefeng Luo, Lianqiang Che, Jiayong Tang

**Affiliations:** ^1^Key Laboratory for Animal Disease-Resistance Nutrition of China Ministry of Education, Animal Nutrition Institute, Sichuan Agricultural University, Chengdu, China; ^2^Department of Veterinary Medicine, University of Perugia, Perugia, Italy; ^3^Dekon Food and Agriculture Group, Chengdu, China

**Keywords:** fermented wheat bran, growth performance, intestinal health, nutrients digestibility, weaned piglet

## Abstract

The objective of this study was to investigate the effects of fermented wheat bran (FWB) on growth performance, nutrient digestibility, serum biochemistry, short-chain fatty acids, and intestinal microbiota of weaned piglets. One hundred twenty-eight weaned piglets were randomly assigned to 4 groups, each with 8 pens and 4 piglets per pen: basal diet group (BD), 5% wheat bran group (5% WB), 5% fermented wheat bran group (5% FWB), and 10% fermented wheat bran group (10% FWB) for a 28-day trial. Results showed that compared to the BD group, the diarrhea rate in the 5% WB group was significantly increased (*p* < 0.05) at d 15–28 and d 1–28. In contrast, at d 15–28 and d 1–28, the diarrhea rates in the 5% FWB and 10% FWB groups were significantly lower than those in the 5% WB group and showed no significant difference compared to the BD group. Moreover, the apparent total tract digestibility (ATTD) of DM, GE, CP, EE, CF and ADF at d 1–14, and EE and NDF at d 15–28 in the 5% FWB group were significantly improved compared to the 5% WB group (*p* < 0.05). However, only the ATTD of CP, EE and CF at d 1–14 in the 10% FWB group were significantly higher than those in the 5% WB group (*p* < 0.01). Compared to the BD group, the pH of cecum chyme and serum urea nitrogen content in the 5% FWB and 10% FWB groups were significantly reduced (*p* < 0.05), and those in the 10% FWB group were significantly lower than those in the 5% WB group (*p* < 0.01). The propionic acid content of cecum chyme in the 5% FWB and 10% FWB groups, and butyric acid content in the 10% FWB group were significantly higher than those in the BD group (*p* < 0.05). LEfSe analysis (LDA score > 3.0) identified 4 species, 6 species of Proteobacteria, 2 species, and 9 species that were enriched in the BD, 5% WB, 5%F WB and 10%F WB groups, respectively. Additionally, *Dialister*, *Prevotellaceae_NK3B31_group*, *Mitsuokella*, *Succinivibrio*, and *Prevotella* were significantly and positively correlated with the concentrations of valeric acid, propionic acid, and acetic acid (*p* < 0.05). In conclusion, 10% FWB supplementation in weaned piglet diets did not affect growth performance, it reduced the diarrhea rate compared to the 5% WB group, potentially due to enhanced nutrient digestibility, elevated SCFAs levels, and shifts in microbial composition.

## 1 Introduction

In recent years, the shortage of corn and soybean meal has severely restricted the rapid development of China’s livestock and poultry breeding, necessitating urgent exploration of unconventional feed materials to address this situation. China possesses abundant unconventional feed resources, such as rapeseed meal, cottonseed meal, rice bran, wheat bran (WB), etc. WB is a by-product of wheat milling, remaining after the extraction of flour and germ. As the world’s largest wheat producer and consumer, China generates 20–30 million tons of WB annually. This by-product is nutritionally dense, containing approximately 15% crude protein (CP), substantial levels of B vitamins (1), and 35–60% dietary fiber (DF) (2, 3). WB is widely used in pig diets and offers multiple benefits, such as improving intestinal health in piglets when supplemented at ~5%, increasing average daily feed intake (4, 5) (ADFI) and enhancing gut microbiota in sows when supplemented with 18% during gestation (6). However, the use of WB in weaned piglet’s diet is limited due to its inferior amino acid profile compared to soybean meal (7), low energy value, high crude fiber (CF) content, antinutritional factors such as phytate, and poor palatability (8, 9). Additionally, WB contains up to 46% non-starch polysaccharides (NSP) (10) and 5% phytic acid, which may significantly impede digestion and absorption in piglets (11, 12).

Weaned piglets have underdeveloped digestive organs, limited organ volume, and insufficient digestive enzymes secretion capacity, making them highly susceptible to weaning stress due to physiological and environmental challenges (13). The digestive capacity of the gastrointestinal tract improves with age. Weaning leads to reduced protease activity in piglets, with typically recover within two weeks post-weaning (14, 15). The low water-holding capacity of WB increases intestinal chyme viscosity in piglets. Supplementation with 5% WB in a fiber-free diet has been shown to exacerbate piglet diarrhea (16, 17). Furthermore, diets containing 20% WB have been found to reduce nutrient digestibility in growing pigs (18).

Microbial fermentation is a widely used method for enhancing the nutritional value of fibrous materials. *Saccharomyces cerevisiae* contains a high bacterial protein content (40–80%) and generates free nucleotides and amino acids during the fermentation, thereby increasing the CP content of the substrate (19, 20). *Lactobacillus* fermentation produces lactic acid, which enhances substrate palatability while inhibiting harmful bacterial colonization (21). *Bacillus subtilis* secretes proteases and cellulases, and its metabolically active spores can reduce intestinal oxygen levels, thereby suppressing harmful bacteria growth (22). Fermented feed components have health-promoting properties as a source of probiotic microbes, digestive enzymes, and antioxidant compounds (23). Mixed fungi-fermented WB can increase the soluble dietary fiber (SDF) content from 5.6 to 13.4% (3), improve the intestinal flora of piglets and enhance their immune function (24). When enzymes are used in combination with microbial fermentation, the essential amino acid content and nutritional value of fermented wheat bran (FWB) become significantly higher than those of unfermented WB (25, 26). However, there is a lack of research on the application of FWB in weaned piglets. Therefore, the purpose of this study was to explore the effects of FWB on growth performance, nutrient digestibility, and intestinal microbiota of weaned piglets.

## 2 Materials and methods

### 2.1 Animal ethics

The experiment was conducted in accordance with the recommendations of “Laboratory Animal-Guideline of Welfare and Ethics of China (GB/ T 35892-2018)” and approved by Institutional Animal Care and Use Committee of Sichuan Agricultural University.

### 2.2 Materials and diet

Wheat bran was obtained from a commercial company (Chengdu Xiongjian Powder Industry Co., Ltd., Chengdu, Sichuan, China), and stored in dry conditions. The fermentation process of FWB was as follows: Based on the weight of WB, 0.5 times (w/v) the volume of water containing cellulase at 200 U/mL was added, and the mixture was thoroughly mixed and allowed to undergo enzymatic hydrolysis at room temperature for 24 h. Then, the bacterial solution equivalent to 0.5 times the weight of WB (w/v) was added, which contained amylase at 1000 U/mL, 8% (NH_4_)_2_SO_4_, and a mixture of *Candida utilis* BNCC 336517, *Lactobacillus plantarum* CGMCC 1.12934, and *Bacillus subtilis* CICC 21095 in a ratio of 3:2:2, resulting in a total bacterial count of 3 × 10^9^ CFU/kg WB. The mixture was stirred again and fermented at 34°C for 3 days, then stored at 4°C for later use. The nutrient composition of wheat bran before and after fermentation was shown in Table 1.

**Table 1:** The nutrient levels of wheat bran before and after fermentation (as air-dry matter basis, %).

Items	WB	FWB
Ether extract	3.75	4.51
Crude protein	19.55	24.62
True protein	15.82	16.84
Acid-soluble protein	2.47	10.75
Crude fiber	12.36	11.41
Neutral detergent fiber	45.29	39.80
Acid detergent fiber	13.50	12.49
Soluble dietary fiber	8.68	11.96
Insoluble dietary fiber	62.34	56.74

WB, wheat bran; FWB, fermented wheat bran.

The diets were supplemented with minerals and vitamins to meet or exceed the requirements for piglets (body weight, 5⁓7 kg and 7⁓11 kg) according to the NRC (2012). The ingredients and nutrient compositions of the diets were reported in Table 2.

**Table 2:** Formulation and chemical compositions of diets (as air-dried fed basis, %)^a^.

Ingredients	Treatments							
	1–14 d				15–28 d			
	BD	5% WB	5% FWB	10% FWB	BD	5% WB	5% FWB	10% FWB
Corn	51.58	46.75	47.24	43.02	57.54	52.80	53.16	49.09
Soybean meal	8.00	6.68	6.30	4.60	14.00	12.68	12.35	10.60
Low protein whey powder	10.00	10.00	10.00	10.00	6.00	6.00	6.00	6.00
Soy protein concentrate	6.00	6.00	6.00	6.00	4.00	4.00	4.00	4.00
Extruded soybean	8.00	8.00	8.00	8.00	6.00	6.00	6.00	6.00
WB	-	5.00	-	-	-	5.00	-	-
FWB	-	-	5.00	10.00	-	-	5.00	10.00
Whole milk powder	5.00	5.00	5.00	5.00	2.00	2.00	2.00	2.00
Fish meal	4.00	4.00	4.00	4.00	2.00	2.00	2.00	2.00
Soybean oil	1.00	2.10	2.00	2.90	2.00	3.00	3.00	3.80
Sucrose	2.00	2.00	2.00	2.00	2.00	2.00	2.00	2.00
L-lysine HCl (78%)	0.73	0.76	0.76	0.79	0.67	0.70	0.70	0.73
DL-Methionine (99%)	0.28	0.30	0.30	0.30	0.25	0.26	0.25	0.26
L-Threonine (99%)	0.28	0.29	0.28	0.29	0.23	0.25	0.23	0.24
L-Tryptophan (99%)	0.09	0.10	0.10	0.10	0.07	0.07	0.07	0.08
Choline chloride (50%)	0.16	0.16	0.16	0.16	0.16	0.16	0.16	0.16
Limestone	0.38	0.40	0.40	0.44	0.48	0.52	0.52	0.54
CaHPO_3_	0.94	0.90	0.90	0.84	1.24	1.20	1.20	1.14
NaCl	0.51	0.51	0.51	0.51	0.51	0.51	0.51	0.51
Zinc oxide (75%)	0.20	0.20	0.20	0.20	-	-	--	
Acidifiers	0.50	0.50	0.50	0.50	0.50	0.50	0.50	0.50
Vitamin-mineral premix^b^	0.35	0.35	0.35	0.35	0.35	0.35	0.35	0.35
Total	100.00	100.00	100.00	100.00	100.00	100.00	100.00	100.00
Nutrient levels (analyzed values, %)								
Crude protein	19.33	19.21	19.64	19.66	17.54	17.85	17.95	17.89
Dry matter	90.42	90.37	88.99	86.38	88.91	89.02	88.07	86.22
Ether extract	4.75	3.68	4.69	4.95	5.31	3.83	6.40	4.75
Crude fiber	2.62	2.80	4.36	5.16	3.71	4.05	4.72	5.02
Neutral detergent fiber	10.68	11.26	11.22	11.68	10.45	11.89	12.86	13.29
Acid detergent fiber	2.57	3.38	2.85	2.86	3.40	4.06	4.08	4.14
Nutrient levels (calculated values, %)								
Digestible energy (Mcal/kg)	3.59	3.59	3.59	3.59	3.55	3.55	3.55	3.55
Ca	0.85	0.85	0.85	0.85	0.80	0.80	0.80	0.80
AP	0.45	0.45	0.45	0.45	0.40	0.40	0.40	0.40
SID^c^ Lysine	1.50	1.50	1.50	1.50	1.35	1.35	1.35	1.35
SID methionine	0.57	0.59	0.59	0.58	0.51	0.51	0.50	0.51
SID methionine + cysteine	0.82	0.82	0.83	0.82	0.74	0.74	0.74	0.74
SID threonine	0.88	0.88	0.88	0.88	0.79	0.79	0.79	0.79
SID tryptophan	0.28	0.28	0.28	0.28	0.24	0.24	0.24	0.24

a All nutritional requirements in the diets of 1–14 d and 15–28 d were met or exceeded NRC (2012) recommendations for 5–7 kg and 7–11 kg piglets, respectively.

b Provided the following per kg of complete diet: Vitamin A, 15000 IU; Vitamin D_3_, 5,000 IU; Vitamin E, 40 IU; Vitamin K_3_, 5.0 mg; Vitamin B_1_, 5.0 mg; Vitamin B_2_, 12.5 mg; Vitamin B_6_, 6.0 mg; Vitamin B_12_, 0.6 mg; Nicotinamide, 50 mg; D-pantothenic acid, 25.0 mg; Folic acid, 2.5 mg; D-biotin, 2.5 mg; Fe (FeSO_4_·7H_2_O), 100 mg; Cu (CuSO_4_·5H_2_O), 6.0 mg; Zn (ZnSO_4_·H_2_O), 100 mg; Mn (MnSO_4_·H_2_O), 4.0 mg; I (KI), 0.14 mg; Se (Na_2_SeO_3_) 0.3 mg.

c SID: Standardized ileal digestible.

BD, basal diet group without wheat bran; 5% WB, 5% wheat bran group; 5% FWB, 5% fermented wheat bran group; 10% FWB, 10% fermented wheat bran group.

### 2.3 Animals, experiment design and management

A total of 128 crossbred (Duroc × Landrace × Yorkshire) weaned piglets, with an average body weight (BW) of 7.59 ± 0.99 kg, were randomly divided into 4 groups, each with 8 pens and 4 piglets per pen (*n* = 8). The 4 groups included the basal diet group (BD), 5% wheat bran group (5% WB), 5% fermented wheat bran group (5% FWB), and 10% fermented wheat bran group (10% FWB). The addition amount of FWB was calculated based on the weight of WB before fermentation, and the diets were provided in powder form. The piglets were housed in floor pens, the room temperature was maintained at 28 ± 1°C, and they were fed *ad libitum* with free access to water. The trial lasted for 28 days and was divided into two stages: d 1–14 and d 15–28. Compared to the BD group, the 5% FWB and 10% FWB groups contained 10.14 and 20.07% less corn and soybean meal at 1–14 d, and 8.43 and 16.56% less at 15–28 d, respectively. All diets contained 0.3% of Cr_2_O_3_ as an indigestible marker to calculate the apparent total tract digestibility (ATTD) of energy and nutrients.

### 2.4 Growth performance and diarrhea rate

All piglets were weighed on days 0, 14 and 28 after an overnight fast, and their feed intake was recorded. The ADG, ADFI and feed-to-gain ratio (F/G) for each pen were then calculated. The general health of all piglets was checked daily during the experimental period. The diarrhea score was based on previous descriptions: 0, normal; 1, pasty; 2, semi-liquid; and 3, liquid (27). Piglets were considered to have diarrhea when the fecal score was ≥2. The diarrhea rate was calculated as follows: Diarrhea rate (%) = (total number of diarrhea piglets × days of diarrhea) / (total number of piglets × days) × 100 (27).

### 2.5 Samples collection

Approximately 500 g of raw WB, FWB, and each group’s diet at each stage were collected and stored at −20°C for analysis. Fresh fecal samples per pen were collected from days 12 to 14 and 26 to 28. Then, 10 mL of 5% H_2_SO_4_ solution was added to each 100 g of fresh fecal sample to fix excreta nitrogen. All samples were then dried at 65°C for 72 h and finely ground for ATTD analysis.

On day 28, after an overnight fast, 6 piglets from each group (*n* = 6) with the average BW from each pen were selected for sample collection. Blood samples (8 mL) were collected from the vena cava into anticoagulant-free tubes and kept at room temperature for 0.5 h. After centrifugation (3,500 × g for 15 min at 4°C), the supernatant (serum) was collected and stored at −20°C for later analysis. The same piglets were then anesthetized with a lethal injection of sodium pentobarbital (200 mg/kg BW) and slaughtered immediately. After opening the abdomen, the tissues of the jejunum, colon, cecum and gastric were quickly removed. The contents of colon, cecum and gastric were then transferred to sterile beakers, and the pH values were measured using a pH meter (FE-28, Mettler Toledo, Switzerland). The chyme from the middle cecum was collected into sterile tubes, placed in liquid nitrogen, and stored at −80°C for microbial analysis. A 10 cm section of the middle jejunum was removed, emptied, and washed with normal saline. A 2 cm section was then cut and fixed in 4% paraformaldehyde solution for histological analysis.

### 2.6 Physicochemical characteristics analyses

The samples of WB, FWB, feed, and feces were dried at 65°C for 72 h, regained moisture for 24 h at room temperature, and then ground and analyzed in duplicate. The WB and FWB samples were placed on double-sided adhesive tape, fixed onto the sample holder of a scanning electron microscope (SEM, Aztec X-Max80, UK), and scanned using ion sputtering to observe microstructural changes. Dry matter (DM) and ether extract (EE) were determined using AOAC method 930.15 (2019) and 920.39 (2019) (28), respectively. Gross energy (GE) was measured using an oxygen bomb calorimeter (Model 6,400, Parr Instrument Company, Moline, IL, United States). N content was determined using AOAC method 990.03 (2019) (28) on a Kjeldahl K-360 (Buchi Corp., Flawil, Switzerland), and CP was calculated as N × 6.25. True protein (TP) content of the WB and FWB was determined by the method of Saavedra-Jiménez (29). Acid-soluble protein (ASP) content in the WB and FWB was determined according to China National Standard (30). CF, acid detergent fiber (ADF), and neutral detergent fiber (NDF) contents were measured using the methods of Van Soest et al. (31). SDF and insoluble dietary fiber (IDF) contents were determined using AOAC method 991.43 and 2011.25 (2019), respectively. Cr content was determined using an atomic absorption spectrometer (contrAA700, Jena, Germany) as described by Kemme et al. (32). The ATTD of nutrient was calculated using our previously reported formula: $\mathrm{Digestibility}\;\left(\%\right)=\left(1-\frac{\left({〖Cr〗}_{\left(\mathrm{content in diet}\right)}\times \mathrm{nutrient content in fecal}\right)}{\left({〖Cr〗}_{\left(\mathrm{content in fecal}\right)}\times \mathrm{nutrient content in diet}\right)}\right)\times 100$ (33).

### 2.7 Serum biochemistry analysis

The concentrations of serum urea nitrogen (SUN), albumin (ALB), alkaline phosphatase (ALP), alanine aminotransferase (ALT), aspartate aminotransferase (AST), and total protein were measured using reagent kits (CH0101051, CH0101002, CH0101203, CH0101201, CH0101202, CH0101008; Maccura, Sichuan, China) with an automatic biochemical analyzer (3,100, HITACHI, Tokyo, Japan). All measurements were performed in duplicate.

### 2.8 Histomorphology measurements

The jejunum samples from 6 pigs per group were fixed in 4% paraformaldehyde solution, dehydrated, and infiltrated with paraffin wax. They were sectioned at 5 μm thickness, stained with hematoxylin and eosin (HE), and examined using a microscope (DM1000, Leica, Germany). Villus height (VH) and crypt depth (CD) were measured for at least 10 well-oriented villus and crypt columns at 10 × magnification with Image-Pro plus 6.0 (Media Cybernetics, Maryland, United States). The ratio of villus height to crypt depth (VH/CD) was then calculated.

### 2.9 Short-chain fatty acids analysis

The contents of acetic acid (AA), propionic acid (PA), butyric acid (BA), and valeric acid (VA) were analyzed using a gas chromatograph system (CP-3800, Varian, Palo Alto, USA) after the pretreatment of cecum chyme, as described by our group (33). Briefly, approximately 3 g of cecal chyme was mixed with ultra-pure water in a 1:1 (w/v) ratio and vortexed. After centrifugation at 10,000 × g for 15 min, 1 mL of supernatant was mixed with 0.2 mL of 25% metaphosphoric acid and 23 μL of 210 mmol/L crotonic acid, then incubated at 4°C for 30 min. Following centrifugation at 8,000 × g for 10 min, 0.3 mL of supernatant was mixed with 0.9 mL methanol (1:3, v/v), and centrifuged at 8,000 × g for 5 min. The final supernatant was filtered through a 0.22 μm membrane and analyzed by gas chromatograph.

### 2.10 Microbial analysis

Cecum chyme samples were analyzed using the 16S rRNA method (33). Briefly, after thawing, 0.25 g of chyme was uniformly sampled, then, the genomic DNA was extracted using the CTAB method. The DNA purity and concentration were assessed using 2% agarose gel electrophoresis and a Nanodrop 2000 (Thermo Fisher Scientific, Waltham, MA, United States). The V4 hypervariable regions of bacterial 16S rRNA genes were amplified using primers 515F (5′-GTGCCAGCMGCCGCGGTAA-3′) and 806R (5′-GGACTACHVGGGTWTCTAAT-3′). Pyrosequencing of bacterial 16S rDNA was performed on the Illumina NovaSeq platform to generate 250 bp paired-end reads. Initial Operational Taxonomic Units (OTUs) were obtained using the DADA2 or deblur module in QIIME2 for denoising. OTUs with an abundance of less than 5 were removed. Species annotations were performed using QIIME2 software. The sequences were submitted to NCBI’s Sequence Read Archive for open access (PRJNA1247028).

### 2.11 Statistical analysis

All data were first tested for normal distribution using the Descriptive Statistics (explore) module in SPSS 27.0 software. For normally distributed data, one-way ANOVA was used to analyze group differences, and Duncan’s multiple range test assessed variance homogeneity between groups. Data were expressed as the mean with pooled standard error (SE). Differences among the four groups were considered statistically significant at *p* < 0.05, whereas *p*-values between 0.05 and 0.10 were considered trends.

For the microbiota data, all indices were calculated using QIIME (Version 1.7.0) and displayed using R software (Version 2.15.3). One-way ANOVA was performed to identify significantly different species at each taxonomic level (Phylum and Genus). LEfSe analysis (LDA score threshold: 3) was performed using LEfSe software (Version 1.0). Spearman correlation analysis was conducted to evaluate the relationship between SCFAs and microorganisms, obtaining correlation and significance values.

## 3 Results

### 3.1 Physicochemical characteristics of FWB

**Figure 1:**
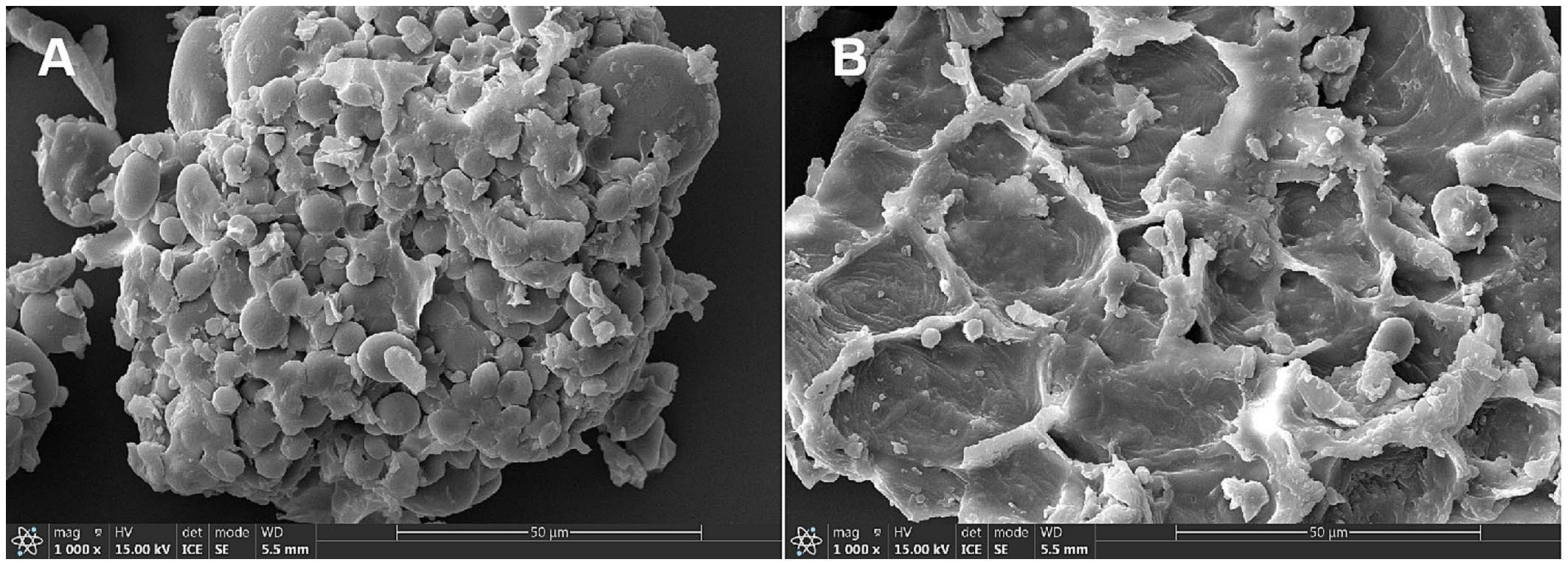
Surface structure of wheat bran **(A)** and fermented wheat bran **(B)**. Scanning electron microscope images at × 1,000-fold magnification.

Compared with WB, FWB increased EE, CP, TP, ASP and SDF by 20.27, 25.93, 6.45, 335.22 and 37.79%, respectively. Meanwhile CF, NDF, ADF, and IDF levels decreased by 7.69, 12.12, 7.48, and 8.98%, respectively (Table 1). The morphological characteristics of WB and FWB were observed under 1,000× SEM, with results presented in Figure 1. The apparent structure of WB was complete, dense, and regular (Figure 1A). After fermentation, the tearing area and crushing structure of FWB were significantly increased compared to before fermentation (Figure 1B).

### 3.2 Growth performance

As shown in Table 3, BW, ADFI, ADF, and F/G did not differ among the BD, 5% WB, 5% FWB, and 10% FWB groups (*p* > 0.05). Compared to the BD group, the diarrhea rate was significantly increased at 15–28 d and 1–28 d (*p* < 0.05) and tended to increase at 1–14 d (*p* = 0.07) in the 5% WB group. However, compared to 5% WB group, the diarrhea rate in the 5% FWB and 10% FWB groups was significantly reduced at 15–28 d and 1–28 d (*p* < 0.05). However, there was no significant difference in the diarrhea rate between the 5% FWB and 10% FWB groups.

**Table 3:** Effects of FWB on growth performance and diarrhea rate of weaned piglets (*n* = 8).

Item	BD	5% WB	5% FWB	10% FWB	SE	*P*-value
Body weight, kg						
0 d	7.57	7.59	7.61.	7.59	0.03	0.97
14 d	10.53	10.87	10.49	10.59	0.07	0.23
28 d	16.67	17.02	16.57	16.90	0.13	0.64
Average daily feed intake, g/d						
1–14 d	305.49	315.85	295.81	294.55	4.16	0.24
15–28 d	702.01	702.70	693.39	703.06	8.85	0.98
1–28 d	503.75	509.28	494.60	498.81	5.93	0.85
Average daily gain, g/d						
1–14 d	211.05	234.82	205.58	214.73	4.46	0.09
15–28 d	438.84	438.84	434.24	450.34	5.92	0.82
1–28 d	324.95	336.83	319.91	332.54	4.55	0.58
Feed/gain ratio, g/g						
1–14 d	1.46	1.35	1.44	1.38	0.02	0.15
15–28 d	1.60	1.60	1.60	1.57	0.01	0.78
1–28 d	1.55	1.51	1.55	1.51	0.01	0.30
Diarrhea rate, %						
1–14 d	7.14	10.49	4.24	5.58	0.89	0.07
15–28 d	2.46^b^	5.58^a^	3.35^b^	3.13^b^	0.40	0.03
1–28 d	4.80^b^	8.04^a^	3.80^b^	4.35^b^	0.56	0.02

BD, basal diet group without wheat bran; 5% WB, 5% wheat bran group; 5% FWB, 5% fermented wheat bran group; 10% FWB, 10% fermented wheat bran group. ab Mean values within a row with different letters differ significantly (*P* < 0.05).

### 3.3 Apparent total tract digestibility

In the first phase (1–14 d), the ATTD of DM, GE, EE, CF, and NDF in the 5% WB group was significantly lower than that in the BD group (*p* < 0.05). However, compared with the 5% WB group, the ATTD of DM, CP, GE, EE, CF, NDF, and ADF in the 5% FWB group was significantly increased (*p* < 0.05). Notably, the ATTD of CP, CF, and ADF in the 5% FWB group was significantly higher than that in the BD group. Furthermore, the ATTD of CF in the 10% FWB group was significantly higher than that in both the BD and 5% WB groups (*p* < 0.01) (Table 4).

**Table 4:** Effects of FWB on apparent total tract digestibility of weaned piglets (*n* = 8).

Nutrients	BD	5% WB	5% FWB	10% FWB	SE	*P*-value
1–14 d						
DM, %	85.07^ab^	82.12^c^	85.75^a^	82.77^bc^	0.49	0.01
CP, %	76.79^bc^	74.12^c^	81.41^a^	79.29^ab^	0.81	< 0.01
GE, %	84.54^ab^	81.96^c^	85.93^a^	82.73^bc^	0.49	0.01
EE, %	73.72^a^	62.58^b^	75.36^a^	73.21^a^	1.54	< 0.01
CF, %	62.21^b^	53.70^c^	75.22^a^	69.56^a^	1.80	< 0.01
NDF, %	63.25^ab^	53.89^c^	65.04^a^	57.99^bc^	1.27	< 0.01
ADF, %	37.29^b^	37.18^b^	49.11^a^	34.15^b^	1.97	0.03
15–28 d						
DM, %	75.37^b^	78.74^a^	76.38^b^	72.69^c^	0.53	< 0.01
CP, %	62.75^b^	71.48^a^	69.47^a^	68.71^a^	0.77	< 0.01
GE, %	75.43^b^	79.00^a^	77.75^a^	73.62^b^	0.51	< 0.01
EE, %	65.59^b^	61.24^b^	78.78^a^	65.84^b^	1.62	< 0.01
CF, %	46.67^a^	44.60^a^	49.28^a^	38.56^b^	1.21	0.01
NDF, %	30.68^c^	39.09^b^	46.26^a^	27.25^c^	1.74	< 0.01
ADF, %	22.94	30.36	34.88	26.41	1.63	0.05

^abc^ Mean values within a row with different letters differ significantly (*P* < 0.05). BD, basal diet group without wheat bran; 5% WB, 5% wheat bran group; 5% FWB, 5% fermented wheat bran group; 10% FWB, 10% fermented wheat bran group; DM, dry matter; CP, crude protein; GE, gross energy; EE, ether extract; CF, crude fiber; NDF, neutral detergent fiber; ADF, acid detergent fiber.

In the second phase (15–28 d), the ATTD of DM, CP, GE and NDF in the 5% WB group was significantly higher than that in the BD group (*p* < 0.05). However, the ATTD of EE and NDF in the 5% FWB group was significantly increased (*p* < 0.05) compared to the 5% WB group. Moreover, the ATTD of CP, GE, EE, and NDF in the 5% FWB group was significantly higher than that in the BD group (*p* < 0.05). In the 10% FWB group, the ATTD of CP was significantly higher, while the ATTD of DM and CF were significantly lower than that in the BD group (*p* < 0.01) (Table 4).

### 3.4 Serum biochemical parameters

Compared to the BD group, the SUN levels in the 5% FWB and 10% FWB groups were significantly decreased, with no significant difference between the two groups. Additionally, the SUN level in the 10% FWB group was significantly lower than that in the 5% WB group (*p* = 0.01). However, neither WB nor FWB diets had no effect (*p* > 0.05) on serum levels of ALB, ALP, ALT, AST, or TP (Table 5).

**Table 5:** Effects of FWB on serum biochemistry of weaned piglets (*n* = 6).

Items	BD	5% WB	5% FWB	10% FWB	SE	*P*-value
Alanine aminotransferase, U/L	70.84	94.61	74.66	76.12	4.19	0.19
Aspartate aminotransferase, U/L	41.03	41.14	44.12	40.29	2.34	0.95
Albumin, g/L	22.83	24.38	26.11	27.01	0.73	0.18
Alkaline phosphatase, U/L	278.00	260.83	302.00	269.17	14.61	0.80
Total protein, g/L	44.88	47.03	49.39	49.37	0.86	0.19
Serum urea nitrogen, mmol/L	2.01^a^	1.82^ab^	1.27^bc^	0.88^c^	0.14	0.01

^abc^ Mean values within a row with different letters differ significantly (*P* < 0.05). BD, basal diet group without wheat bran; 5% WB, 5% wheat bran group; 5% FWB, 5% fermented wheat bran group; 10% FWB, 10% fermented wheat bran group.

### 3.5 The pH of intestinal and gastric chyme

**Figure 2:**
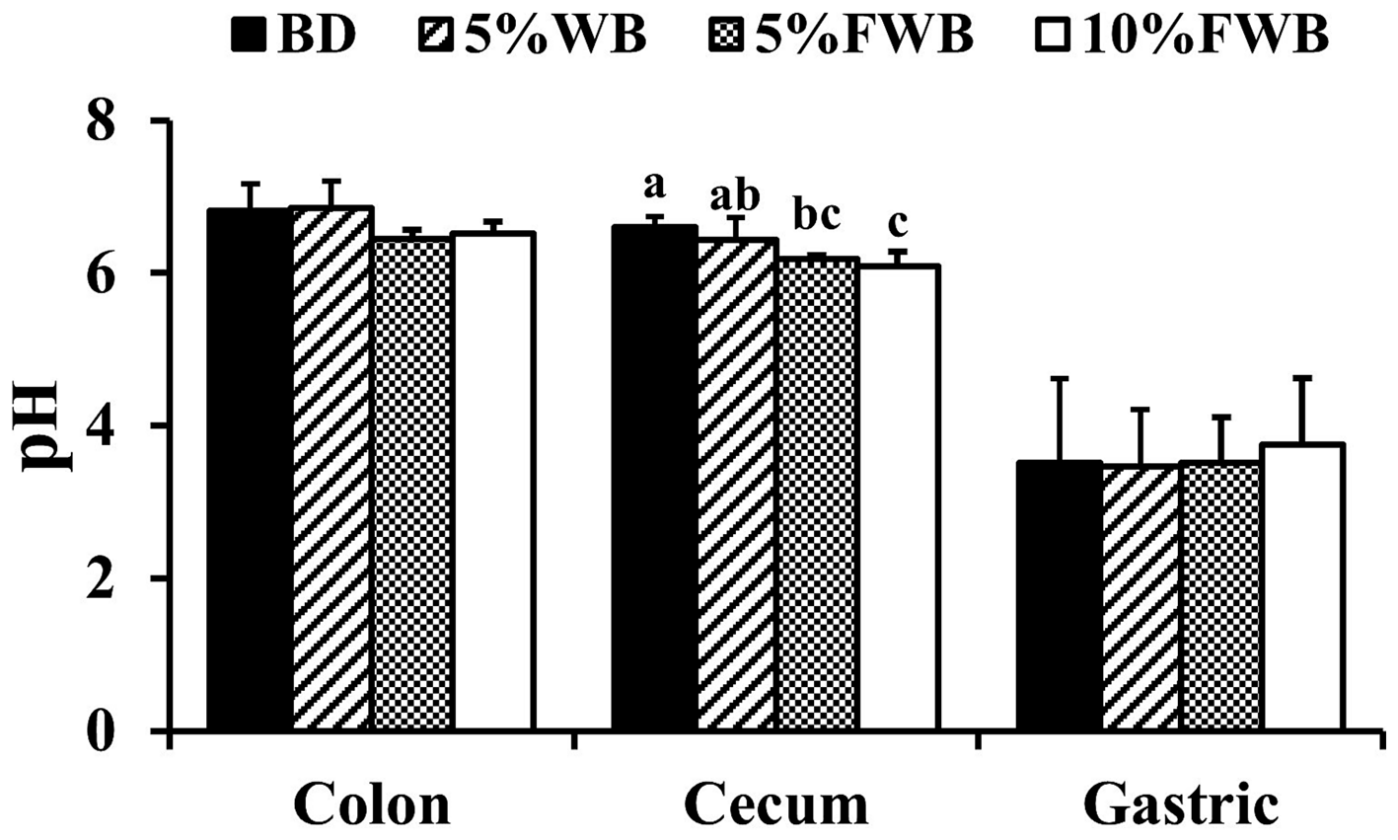
Effects of FWB on intestinal chyme and gastric contents pH of weaned piglets. Mean values with different letters on vertical bars differ significantly (*p* < 0.05). BD, Basal diet group; 5% WB, 5% Wheat bran group; 5% FWB, 5% Fermented wheat bran group; 10% FWB, 10% Fermented wheat bran group.

In cecum chyme, compared to the BD group, the pH of the 5% WB group remained unchanged (*p* > 0.05), but the pH in the 5% FWB and 10% FWB groups was significantly decreased (*p* < 0.01) (Figure 2). However, there was no significant difference in the pH of colonic chyme and gastric contents among the four groups.

### 3.6 Intestinal morphology and short-chain fatty acids

According to the results of the histological analysis, there was no significant difference in jejunum morphology among the four groups of weaned piglets (*p* > 0.05) (Table 6). Compared to the BD group, the PA content in cecum chyme was significantly increased (*p* < 0.05) in the 5% FWB and 10% FWB groups, with no significant difference between these two groups. Additionally, the BA content in the 10% FWB group was significantly higher than in the BD group (*p* < 0.05), with no significant difference compared to the 5% WB and 5% FWB groups (Table 7).

**Table 6:** Effects of FWB on jejunum morphology of weaned piglets (*n* = 6).

Items	BD	5% WB	5% FWB	10% FWB	SE	*P*-value
Villus height, μm	359.44	388.94	433.76	422.32	12.78	0.15
Crypt depth, μm	192.59	219.65	189.13	185.35	8.99	0.55
Villus height/crypt depth	2.04	1.95	2.38	2.38	0.09	0.22

BD, basal diet group without wheat bran; 5% WB, 5% wheat bran group; 5% FWB, 5% fermented wheat bran group; 10% FWB, 10% fermented wheat bran group.

**Table 7:** Effects of FWB on short-chain fatty acids in piglet cecum chyme (*n* = 6).

Item	BD	5% WB	5% FWB	10% FWB	SE	*P*-value
Acetic acid, mg/g	5.25	4.81	5.00	4.71	0.13	0.51
Propionic acid, mg/g	2.31^b^	2.29^b^	2.85^ab^	3.33^a^	0.15	0.03
Isobutyric acid, mg/g	0.04	0.05	0.02	0.02	0.01	0.64
Butyric acid, mg/g	0.76^b^	0.95^ab^	1.01^ab^	1.13^a^	0.05	0.03
Isovaleric acid, mg/g	0.14^a^	0.09^bc^	0.10^b^	0.06^c^	0.01	< 0.01
Valeric acid, mg/g	0.19	0.21	0.25	0.25	0.02	0.34

^abc^ Mean values within a row with different letters differ significantly (*P* < 0.05). BD, basal diet group; 5% WB, 5% wheat bran group; 5% FWB, 5% fermented wheat bran group; 10% FWB, 10% fermented wheat bran group.

### 3.7 Cecum microbiota composition

Dietary supplementation with 10% FWB tended to reduce the Chao 1 index (*p* = 0.07) in cecum chyme compared to the BD, 5% WB, 5% FWB groups (Table 8). There was no significant difference in the *α*-diversity indices of Shannon and Simpson among the four groups (*p* > 0.05).

**Table 8:** Effects of FWB on α-diversity of cecum microorganisms of weaned piglets (*n* = 6).

Items	BD	5% WB	5% FWB	10% FWB	SE	*P*-value
Shannon	6.59	6.54	6.59	5.84	0.14	0.13
Simpson	0.97	0.97	0.97	0.95	0.01	0.22
Chao 1	629.38	630.22	611.62	463.98	26.88	0.07

BD, basal diet group without wheat bran; 5% WB, 5% wheat bran group; 5% FWB, 5% fermented wheat bran group; 10% FWB, 10% fermented wheat bran group.

**Figure 3:**
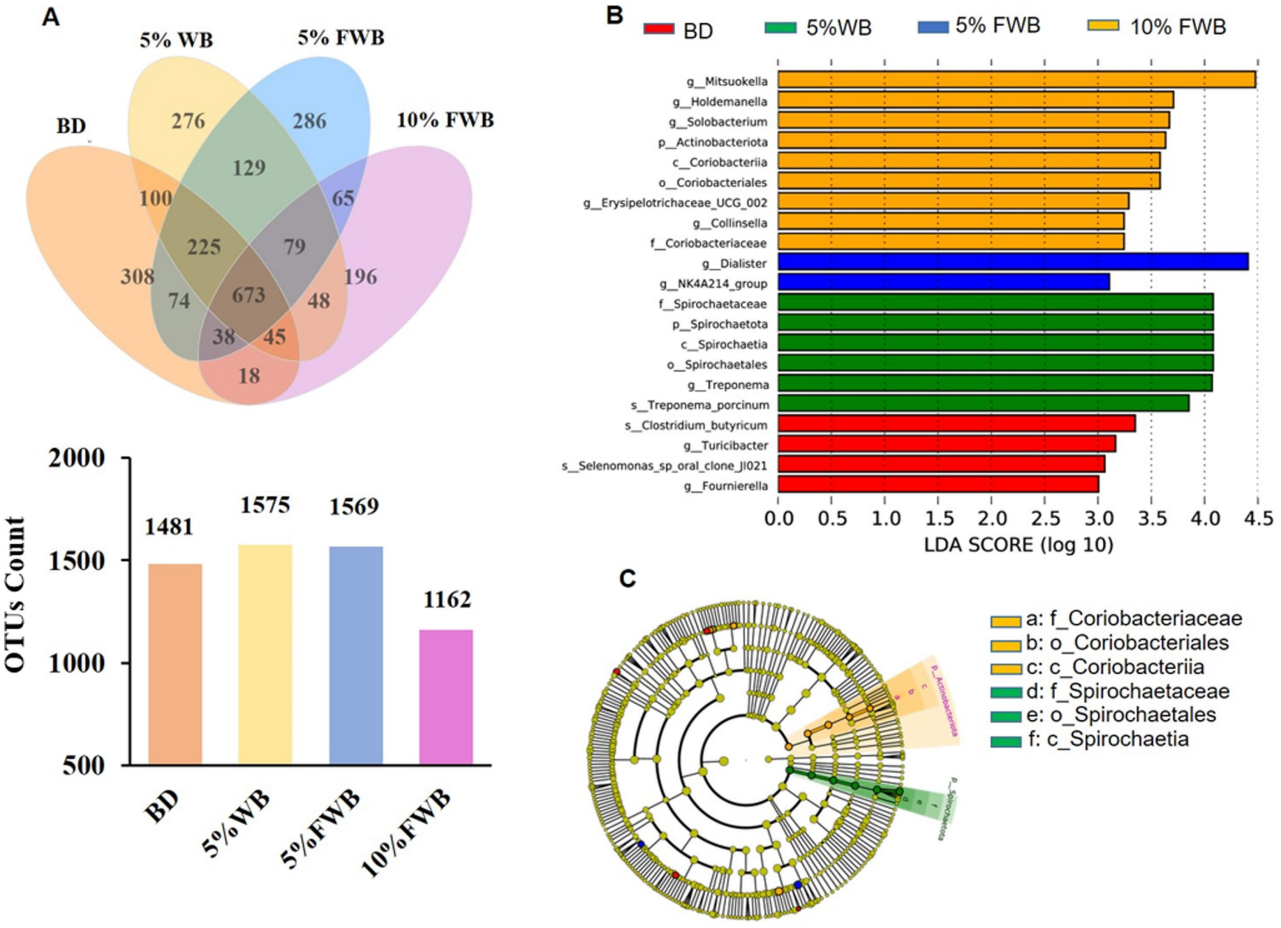
Effects of FWB on cecum chyme microbial characteristics of piglets on day 28 post-weaning (*n* = 6). **(A)** The unique and shared OTUs in the each group; **(B)** LDA scores show the significant bacterial differences among the groups (*p* < 0.05, LDA score > 3.0). **(C)** Cladogram using the LEfSe method shows the phylogenetic distribution of the cecum microbes among the groups. FWB, fermented wheat bran; LDA, Linear discriminant analysis; OTUs, Operational taxonomic units.

**Figure 4:**
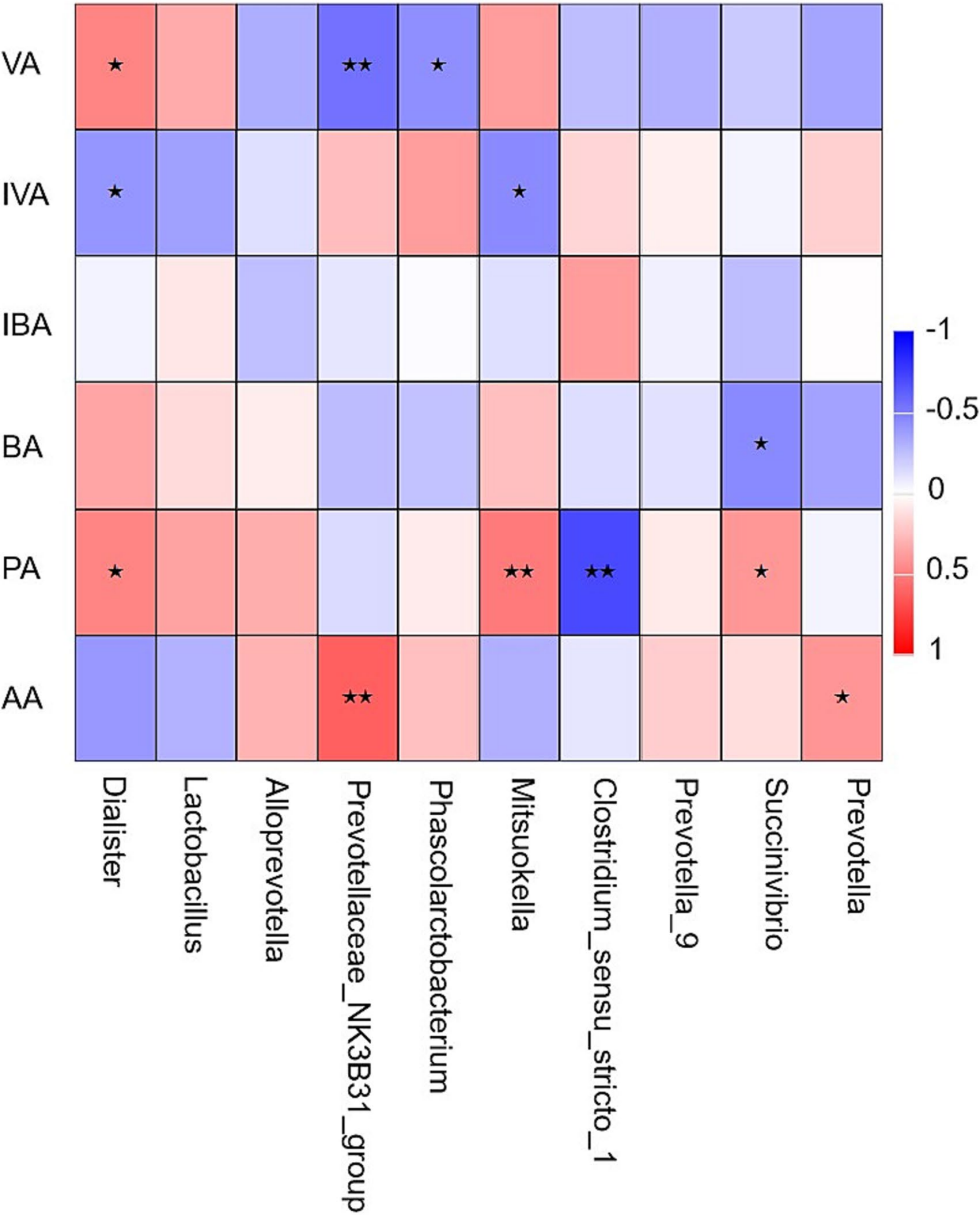
Heat map of the correlation analysis between short-chain fatty acids and microorganisms at genus level. VA, valeric acid; IVA, isovaleric acid; IBA, isobutyric acid; BA, butyric acid; PA, propionic acid; AA, acetic acid. * Indicates a significant correlation between microbes and short-chain fatty acids (*P* < 0.05). ** Indicates an extremely significant correlation between microbes and short-chain fatty acids (*P* < 0.01).

The microbial characteristics of cecum chyme in piglets on d 28 post-weaning was presented in Figure 3. A total of 598,713, 615,078, 619,874, and 600,325 high-quality sequences were obtained from cecum chyme samples in the BD, 5% WB, 5% FWB, and 10% FWB groups, respectively. The Venn diagram shows 1,481, 1,575, 1,569 and 1,162 OTUs in the BD, 5% WB, 5% FWB, and 10% FWB groups, respectively (Figure 3A). The four groups shared 673 OTUs, with the BD, 5% WB, 5% FWB, and 10%FWB groups having 308, 276, 286, and 196 unique OTUs, respectively (Figure 4). Figures 3B,C showed species with significant differences among the 4 groups when the LDA score was greater than 3.0. LEfSe analysis identified 4 species (s_*Clostridium_butyricum*, g_*Turicibacter*, s_*Selenomonas_sp_oral_clone_JI021*, g_*Fournierella*), 6 species of Proteobacteria (f_*Spirochaetaceae*, p_Spirochaetota, c_Spirochaetia, o_Spirochaetales, g_*Treponema*, s_*Treponema_porcinums*), 2 species (g_*NK4A214_group* and g_*Dialister*), and 9 species (g_*Mitsuokella*, g_*Holdemanella*, g_*Solobacterium*, p_Actinobacteriota, c_Coriobacteriia, o_Coriobacteriales, g_*Erysipelotrichaceae_UCG_002*, g_*Collinsella*, f_*Coriobacteriaceae*) enriched in the BD, 5% WB, 5% FWB, and 10% FWB groups, respectively.

Ten bacterial genera with relative abundance greater than 1% at the genus level were analyzed for Spearman correlation with SCFAs in cecum chyme. As shown in Figure 4, the concentration of VA was significantly positively correlated with *Dialister* (r = 0.48, *p* < 0.05) and significantly negatively correlated with *Prevotellaceae_NK3B31_group* (r = −0.55, *p* < 0.01) and *Phascolarctobacterium* (r = −0.44, *p* < 0.05). The concentration of isovaleric acid (IVA) was significantly negatively correlated with *Dialister* and *Mitsuokella* (r = −0.41, *p* < 0.05; r = −0.46, *p* < 0.05). *Succinivibrio* was significantly negatively correlated with BA concentration (r = −0.46, *p* < 0.05) and significantly positively correlated with PA concentration (r = 0.41, *p* < 0.05). The concentration of PA was significantly positively correlated with *Dialister* (r = 0.48, *p* < 0.05) and *Mitsuokella* (r = 0.52, *p* < 0.01) and significantly negatively correlated with *Clostridium_sensu_stricto_1* (r = −0.71, *p* < 0.01). *Prevotellaceae_NK3B31_group* (r = 0.62, *p* < 0.01) and *Prevotella* were significantly positively correlated with AA concentration (r = 0.41, *p* < 0.05).

## 4 Discussion

Wheat bran has not been widely used because it contains a high content of anti-nutrients (such as CF), which negatively affect growth performance and nutrient digestibility in weaned piglets (24). After solid-state fermentation (SSF), CF, NDF and ADF content of FWB were significantly reduced, while the SDF and protein quality were improved (3, 24). This is consistent with the findings of this study. The original dense structure of WB developed obvious tearing surface and pore structure after SSF (24, 34). This looser structure is more favorable for microorganisms to access and fully ferment the nutrients (34).

Piglets have limited tolerance to CF, and excessive levels can reduce their growth performance (35). Due to its large amount of fiber and strong resistance to natural intestinal degradation and digestion, WB is largely used in animal feed, but it is not used to feed young animals such as piglets (36). Thus, dietary supplementation of WB could not improve the growth performance of pigs (24). Similarly, replacing 7.2% of corn in the diet with 8% FWB did not significantly affect the growth performance of weaned piglets (24). In this study, dietary supplementation with 5% or 10% FWB did not significantly affect the growth performance of piglets, consistent with previous studies (25), indicating that FWB can feasibly replace some soybean meal and corn in piglet diets. However, dietary supplementation of 5% FWB significantly increased ADG and F/G in growing-finishing pigs (37), which should be related to the well-developed intestinal system of pigs. Additionally, the high content of NSP in WB limits its use in piglet feed, as piglets often suffer stress-induced diarrhea due to the inadequate development of their intestines and microbiota (38). The increase in NSP concentration increases the viscosity of the chyme and digestive fluid, resulting in undigested chyme entering the colon, where elevated osmotic pressure and water influx lead to diarrhea (16, 39). In this study, dietary supplementation with 5% WB significantly increased the diarrhea rate of piglets, which is consistent with previous study (18). However, the diarrhea rate in the 5% FWB and 10% FWB groups was significantly lower than in the 5% WB group. This is likely related to the fact that FWB has lower CF, NDF, ADF, but higher SDF than WB. SDF is well-known for being largely degraded by microbes in the hindgut of pigs, thus benefiting intestinal homeostasis (39). Additionally, the higher SDF concentration in FWB can slow chyme transit, improve fecal formation, and thereby reduce diarrhea incidence (16, 24).

The high IDF content of WB accelerates gastric emptying and shortens diet retention time in the gastrointestinal tract of piglets (39). However, the gastrointestinal tract of weaned piglets is not capable enough of secreting digestive enzymes, so WB is seldom added to their diets. The current results demonstrated that the ATTD of DM, GE, EE, CF and NDF in the 5% WB group was lower than that in the BD group on d 0–14. However, the ATTD of DM, CP, GE and NDF in the 5% WB group was higher than that in the BD group on d 15–28, but this had no significant effect on the growth performance, which may be related to factors such as energy loss associated with fiber fermentation in the hindgut and shorter experimental period and the treatment replicates, among other factors. Previous studies have shown that the digestibility of CP and EE in the 8% WB group significantly increased compared to the control group at 40 days post-weaning (24). This may be related to the maturation of the piglets’ intestinal development in the later stage of nursery, allowing better secretion of digestive enzymes. Fermented feeds are more palatable (40). The acidic environment enhances intestinal secretion of proteases and other digestive enzymes (17). Additionally, degradation of large-molecule proteins in FWB improves nutrient digestibility in piglets. In this study, the ATTD of DM, CP, GE, EE, CF, NDF, and ADF in the 5% FWB group was higher than in the 5% WB group at 0–14 d. Notably, fiber digestibility in piglets was equal to or better in the 5% FWB and 10% FWB groups compared to the BD group. This is attributed to effective fiber degradation after SSF, indicating FWB did not negatively affect the fiber digestibility in piglets, consistent with a previous study (24), while has found no significant difference in nutrient digestibility between piglets in the 8% WB and 8% FWB groups.

The microbial fermentation process can produce beneficial substances, such as small-size peptides, exoenzymes, vitamins, and organic acids, which can enhance the physiological metabolism of animal (41). Serum biochemical indicators visualize physiological and metabolic functions in animals (42). SUN is an important index reflecting overall protein metabolism (43). The SUN content of growing pigs in the 10% *Aspergillus niger*-fermented canola meal group was found to be significantly reduced by 26.48% compared to the unfermented group (44). In this study, the SUN levels in the 5% FWB and 10% FWB groups were significantly decreased compared to the BD group. This indicates that piglets in the 5% FWB and 10% FWB groups showed greater protein utilization, suggesting that FWB is beneficial for regulating protein digestion and metabolism.

Diarrhea is closely related to small intestinal health, especially the jejunum, which is crucial for nutrient digestion and absorption (38). However, this study found no significant differences in jejunum morphology among the four groups. Additionally, a more acidic intestinal environment benefits piglets’ digestion and absorption and has a positive effect on their intestinal health (45). In this study, the pH of colon and cecum chyme was decreased in the 5% FWB and 10% FWB groups compared to the BD group. The addition of wheat to the diet enhances SDF fermentation in the hindgut, significantly reducing cecal chyme pH in piglets (46), which aligns with the findings of this study. The pH of cecum and colonic chyme serves as an important indicator for evaluating intestinal health in weaned piglets. A lower colon pH would reduce the colonization of enterotoxin-producing bacteria like *Anaplasma* sp. and *Clostridium* sp., beneficial for maintaining intestinal homeostasis in piglets (46).

Probiotic-fermented food or feeds may effectively regulate gut microbiota and its metabolites, such as SCFAs (33). SCFAs are mainly produced in monogastric animals through the fermentation of SDF by beneficial flora like *Lactobacilli* and *Bifidobacteria* in the hindgut (47). They play a crucial role in glycolipid metabolism and intestinal homeostasis in piglets. The rate of microbial fermentation is related to the solubility and viscosity of DF (48), and the loose structure of WB after fermentation enables easier access for microorganisms, accelerating the fermentation rate (24). PA is a substrate for hepatic gluconeogenesis (49). BA is a major energy source for colonic epithelial cells (50). Butyrate can prevent pathogenic microorganisms from attaching to the intestinal mucosa, alleviating intestinal inflammation in *E. coli* infected piglets (51). In this study, PA content in the 5 and 10% FWB groups and BA content in the 10% FWB group were significantly higher than in the BD group. Moreover, SCFAs, as important intermediate products during anaerobic digestion, can inhibit harmful flora, promote the colonization of beneficial flora, and improve intestinal microorganism metabolism in piglets (13). This may explain the decreased diarrhea rate in piglets in this study. *In vitro* digestion of sugarcane polyphenols and fiber significantly decreased IVA, while increasing in BA and total SCFAs (52). These findings are consistent with the significant reduction in cecal chyme IVA content following WB or FWB supplementation observed in our study. As a minor SCFA, IVA production may be reduced by the combined dietary increase of fiber and polyphenols (53, 54), which appear to suppress IVA-producing bacterial metabolism while promoting BA and PA production.

Intestinal microflora plays a crucial role in regulating intestinal health in pigs (55). The Chao 1 index in the 10% FWB group tended to decrease compared to the other groups. This indicates fewer microbial species in the cecum chyme of piglets in the 10% FWB group, likely related to the introduction of *Candida utilis*, *Lactobacillus plantarum*, and *Bacillus subtilis* in FWB. *Lactobacillus* promotes intestinal health in piglets by producing organic acids and bacteriocins, lowering environmental pH and inhibiting harmful bacteria like *E. coli* and *Salmonella* (56). LEfSe analysis showed that the relative abundance of f_*Spirochaetaceae*, p_Spirochaetota, c_Spirochaetia, o_Spirochaetales, g_*Treponema*, s_*Treponema_porcinums* increased in the 5% WB group. Previous studies have found that *Spirochaetes* was significantly and positively correlated with the diarrhea rate in piglets (57). In this study, *Dialister* was enriched in the 5% FWB group, which is consistent with previous reports that enriched SCFAs in piglet feces by *Lactobacillus*-fermented feed, thereby increasing the beneficial effects on piglet intestinal health (58). Dietary yeast supplementations may also promote *Dialister* colonization in the porcine intestinal tract, leading to elevated BA levels (59). Previous studies have shown that *Dialisters* abundance increases significantly by 27 days post-weaning compared to 7 days (60), and the changes in its abundance may be related to the differences in diarrhea among piglets at different stages after weaning. Increased relative abundance of *Dialister* in growing pig’s feces leads to higher total AA and SCFA in the cecum (59). The 10% FWB group showed increased relative abundance of beneficial bacteria like p_Actinobacteriota, g_*Mitsuokella* and g_*Holdemanella*. Higher relative abundance of Actinobacteriota in the cecum of fattening pigs significantly increased intramuscular fat content in the longest dorsal back muscle (61). Moreover, higher abundance of Actinobacteriota can promote BA production (62). *Mitsuokella* can produce fermentation acids and lower the pH to inhibit the growth of *Salmonella Typhimurium* (63), which helps maintain gut health. The increase in relative abundance of Actinobacteriota, *Mitsuokella*, *Holdemanella* and *Dialister* indicates that FWB benefits the colonization of beneficial bacteria in the intestines of weaned piglets, thereby improving their intestinal health, which may be related to its fiber structure (64).

The gut microbiota composition is associated with SCFAs (55). Spearman’s correlation analysis revealed significant positive correlations between the relative abundance of *Dialister*, *Succinivibrio*, *Prevotellaceae_NK3B31_group*, and *Prevotella* and the concentrations of VA, PA, and AA. Higher SDF content in FWB indicates a close relationship between microbial fiber fermentation in the piglet hindgut and SCFAs production. *Prevotella*, prevalent in the pig cecum, produce SCFAs by degrading SDF (65). Similarly, *Succinivibrio* ferments carbohydrates into metabolites like AA and succinic acid (66). Spearman’s correlation analysis indicated that FWB enhanced the colonization of the intestinal tract by genera involved in the metabolism of DF and polysaccharides, increasing the levels of PA and BA, potentially benefiting piglet intestinal health.

## 5 Conclusion

In this study, substituting some soybean meal and corn with FWB in the weaned piglet diet enhanced nutrient digestibility, increased intestinal SCFAs levels, and improved the structure of intestinal microflora, thereby reducing the diarrhea rate. Therefore, this study provides valuable insights into alleviating the shortage of feed resources and supports the application of FWB in weaned piglets.

## Data Availability

The original contributions presented in the study are publicly available. This data can be found at the NCBI with accession number: PRJNA1247028.

## Glossary

AA: Acetic acid
ADF: Acid detergent fiber
ADFI: Average daily feed intake
ADG: Average daily gain
ASP: Acid soluble protein
ATTD: Apparent total tract digestibility
BA: Butyric acid
CF: Crude fiber
CP: Crude protein
DF: Dietary fiber
DM: Dry matter
EE: Ether extract
F/G: Feed/Gain
FWB: Fermented wheat bran
IDF: Insoluble dietary fiber
NDF: Neutral detergent fiber
NSP: Non-starch polysaccharides
OTUs: Operational taxonomic units
PA: Propanoic acid
SCFAs: Short-chain fatty acids
SDF: Soluble dietary fiber
SEM: Scanning electron microscope
TP: True protein
VA: Valeric acid
WB: Wheat bran
